# The impact of “World Health Organization - Government of India guidelines on chronic obstructive pulmonary diseases-2003” on quality of life

**DOI:** 10.4103/0970-2113.56341

**Published:** 2009

**Authors:** Ashok K. Janmeja, Prasanta R. Mohapatra, Mandeep Kumar

**Affiliations:** *Department of Pulmonary Medicine, Government Medical College and Hospital, Chandigarh, India*

**Keywords:** Chronic obstructive pulmonary disease, St. George's respiratory questionnaire, quality of life

## Abstract

**Background:**

The World Health Organization-Government of India (WHO-GOI) Guidelines - 2003 for management of chronic obstructive pulmonary diseases (COPD) is a consensus statement. However, the outcome and impact of its implementation on quality of life (QOL) among COPD patients has not been studied so far.

**Materials and Methods:**

The patients were randomized to intervention group (n = 50) and control group (n = 40). All were treated and followed up for 6 months. A pulmonary physician reviewed patients of both the groups, at least 3 times in 6 months period. St. George's Respiratory Questionnaire was measured at baseline and at 6 months. Patients in control group visited the center on a “need to” basis and were prescribed conventional treatment by the doctor on duty.

**Results:**

Forty-two patients in the intervention group and 32 in the control group completed 3 visits over the period of 6 months and were included in analysis. The severity as per the guidelines was moderate in 74% and severe in 26% in intervention group while it was moderate in 64% and severe in 36% cases in control group. Follow-up QOL scores were significantly better as compared with baseline values (*P* < 0.001). The QOL of the patients treated according to the guidelines were significantly better (*P* < 0.001) than those in the control group with conventional treatment.

**Conclusion:**

The consensus derived recommendations of WHO-GOI Guidelines for COPD-2003 are beneficial for management of COPD patients over conventional management.

## INTRODUCTION

Chronic obstructive pulmonary disease (COPD) is characterized by airflow limitation that is not fully reversible. COPD is a major public health problem worldwide and is expanding throughout with a higher prevalence, morbidity and mortality rate. The World Health Report-2002 listed COPD as the fifth leading cause of death.[1] It currently ranks number 6 in global disease impact scale and is predicted to rise to number 3 by 2020.[1] The prevalence of COPD in India is 5% in males and 2.7% in females, with the male to female ratio of 1.6:1.[2] It translates into approximately 12 million cases in India alone. The incidence and prevalence of COPD is increasing as a result of urban ambient air pollution and indoor exposure concentrations of particulate air pollution.[3–5]

The World Health Organization and Government of India (WHO-GOI) Guidelines-2003 for management of COPD provide management recommendations for optimum practice that include hospital follow-up for those admitted with acute exacerbations and for stable COPD cases involving a number of potential therapeutic interventions.[6]

These guidelines were formulated based on consensus view of experts from all over India during 2002-2003 at the Post Graduate Institute of Medical Education and Research, Chandigarh, under the auspices of WHO and GOI. Health-related quality of life (QOL) has been defined as the functional effect of an illness and its consequent therapy on a patient, as perceived by the patient. QOL is an important domain for measuring the impact of guidelines on management of COPD. However, the outcome of its implementation and their impact on quality of life in COPD patients have not been studied in actual medical practice so far to the best of our knowledge. The disease-specific instruments, such as St. George's Respiratory Questionnaire (SGRQ), have been used to measure health- related QOL in patients with COPD. We had undertaken the study comparing hospital outpatient follow-up according to “WHO-Government of India COPD Guidelines-2003,” with the conventional practice of follow-up care in patients with stable COPD.

## MATERIALS AND METHODS

We included 90 patients with diagnosis of COPD over a 1-year period from April 2006 through 2007 in our study. COPD was diagnosed as per diagnostic criteria of “WHO-Government of India Guidelines-2003 for management of COPD.”[6]

Lung function data were obtained with use of the Spirometer. Lung function was measured before and 15 minutes after administration of 200-μg salbutamol by nebulizations. All spirograms were reviewed by the chest physicians. Data for forced expiratory volume in 1 second (FEV_1_) and forced vital capacity (FVC) were deemed usable and included in this analysis if they fully met the American Thoracic Society (ATS) acceptability criteria and were reproducible to within 200 mL. We included the patients with a prebronchodilator FEV_1_ of <80% of the predicted value, an increase in FEV_1_ with the use of 200 μg of salbutamol of <12% of the predicted value for that patient, and a ratio of prebronchodilator FEV_1_ to FVC ≤0.70.

COPD was diagnosed if patients had progressive symptoms and a FEV_1_ <80% of that predicted with FEV_1_/FVC ratio <0.7. The response to inhaled bronchodilator of more than 12% was taken to discriminate asthma from COPD. The patients with comorbid illness for example – associated or suspected malignancy, tuberculosis, significant cardiac morbidity, any dominant medical condition requiring hospital follow-up, already under outpatient follow-up, already under pulmonary rehabilitation care, mild COPD and those who refused for participation were excluded from study. Patients were randomized to intervention group and control group by simple randomization technique.

After initial recording of demographic data, spirometry including bronchodilator reversibility response and St. George's Respiratory Questionnaire was measured at baseline and three and six months. Numbers of admissions to the hospital, oxygen supplementation, nebulizer prescriptions over six-month periods of follow-up were recorded in all patients. For the SGRQ, a change in four units or more was accepted as significant.[7,8]

The SGRQ is a standardized self-administered airways disease-specific questionnaire that provides an overall measure for quality of life with subscale scores in three areas: Symptom, activity, and impact of disease on daily life. It contains 50 items (covering 76 levels) divided into three subscales: “Symptoms” (8 items), including several respiratory symptoms, their frequency and severity; “Activity” (16 items), concerned with activities that cause or are limited by breathlessness; and “Impacts” (26 items), which covers a range of aspects concerned with social functioning and psychological disturbances resulting from airways disease. Each item in the questionnaire has a weight attached, which provides an estimate of the distress associated with the symptom or state described. A score was calculated for each subscale of the SGRQ and also an overall score was calculated on Microsoft Excel sheet (calculator obtained from P.W. Jones). SGRQ scores range from 0-100, with score 0 indicating no impairment of life quality and 100 indicating maximum disability. The questionnaire has been shown to be reproducible and valid. We adapted the English language of the SGRQ as received and doctors' local language was used while interpreting the questionnaires and aimed to reflect the usual language of the patients.

### Intervention group treatment protocol

A chest physician reviewed the intervention group at least three times in the six month period, that is, at baseline, during the third and sixth month. The spirometry, review of inhaler technique, peak flow diary, ambulatory oxygen assessment, smoking cessation advice, steroid trial, nebulizer usage, review of medication for the addition of long acting B_2_ agonists and theophyllines, advice about nutrition and exercise were made as needed during these visits. Inhaler techniques and compliance were assessed at every visit.

Patients came for regular follow-ups in our outpatient department (OPD) and were given regular treatment strictly according to WHO-GOI COPD Guidelines-2003,[6] as below.

1. Mild COPD - Short acting bronchodilators, when needed
2. Moderate COPD - Regular treatment with one or more bronchodilators
3. Severe COPD - As in moderate COPD plus inhaled corticosteroids, treatment of complications

### Control group treatment protocol

Patients visited our OPD on a “need to” basis and they were not given treatment strictly as per the guidelines in question by the doctors not aware exactly of the guidelines.[6] Drugs used in this group were from among various combinations of oral/inhaled bronchodilators and inhaled/oral corticosteroids depending on the degree of symptom control. Exacerbations were managed appropriately in a similar manner.

The study was approved by our institutional ethics and research committees of Government Medical College and Hospital, Chandigarh, and was conducted in accordance with the Declaration of Helsinki and Good Clinical Practice guidelines.

## RESULTS

The 90 patients included in the study were randomized in to two groups. Fifty patients were placed in the intervention group and 40 patients in the control group. Of these patients, 42 in the intervention group and 32 in the control group completed at least three visits over a period of 6 months and they were included in the data analysis [Table 1].

**Table 1:** Profile of chronic obstructive pulmonary disease patients who were included in the data analysis

	Intervention group	Control group
Gender	Male = 40, Female = 2	Male = 29, Female = 3
Age in years, mean ± SD (range)	60.7 ± 9.92	61.4 ± 9.3
Years of symptoms, mean ± SD (range)	5.14 ± 2.6	5.16 ± 2.92
Smoking history		
Nonsmoker (%)	2 (4.76)	3 (9.38)
Smoker (%)	40 (95.23)	29 (90.62)
FEV_1_ % mean ± SD (range)	51.11 ± 13.6	53.57 ± 9.8
FEV_1_, mean ± SD (range), in L/min	1.27 ± 0.24	1.34 ± 0.29
COPD severity staging		
Moderate (%)	31 (73.8)	20 (62.5)
Severe (%)	11 (26.19)	12 (37.5)

Mean age was 60 years (range 36-80) in intervention group and 61 years (range 35-83) in control group. The ratio of smokers to nonsmokers was 40:2 in intervention group and that 29:3 in control group. According to the severity criteria of “WHO-Government of India Guidelines-2003 for management of COPD,” 74% had moderate and 26% had severe disease in intervention group, while in the control group 64% patients had moderate and 36% were having severe disease.

**Figure 1:**
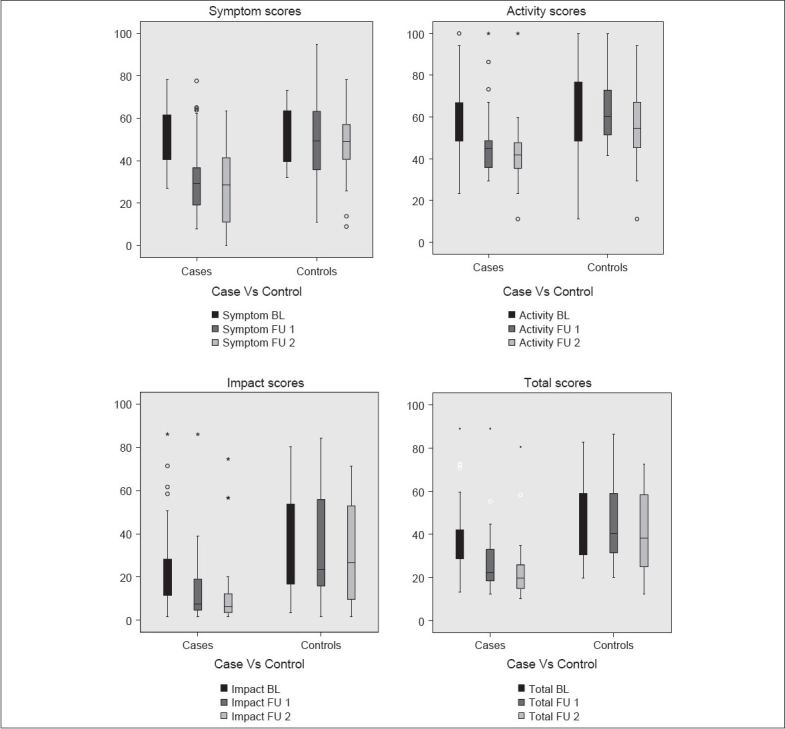
Graphical presentation of SGRQ scores: Symptom, impact, activity, and total score in the control and intervention (cases) groups. (BL = Base line, FU1 = First follow-up after 3 months, FU2 = Second follow-up after 6 months)

There was no significant difference in the baseline quality of life variables in both the groups. Follow-up quality of life scores were significantly better as compared to baseline values (repeated-measures analysis of variance, *P* < 0.001). The quality of life of the patients treated under guidelines management protocol were significantly better (*P* < 0.001) than those in the control group managed with conventional treatment. Graphical presentation of the SGRQ scores: Symptom, impact, activity, and total scores in the control and intervention groups are compared in Figure 1. There was statistically significant (*P* < 0.001) improvement in respiratory QOL scores favoring intervention group patients.

## DISCUSSION

Despite the enormous disease burden of COPD, no study has addressed the value of the WHO-GOI recommendations for active follow-up of the patients. The present study suggests that regular follow-up in the outpatient department accompanied by aggressive implementation of “WHO-Government of India Guidelines-2003 for management of COPD” produces disease-specific improvements in quality of life indicators. The observed change in the SGRQ could be attributed to the effect of giving treatment strictly according to the WHO-GOI guidelines. There were some patients who could not be enrolled mainly due to the disease severity and they could not ethically be randomized to the control group.

This study is worth mentioning for two reasons. Firstly, it objectively justifies the consensus derived recommendations of “WHO-Government of India Guidelines-2003 for management of COPD.” Second, it highlights the surprising paucity of literature on this important area. Larger multicentric studies are needed to confirm our findings and to identify the individual interventions that may account for a change in quality of life indices and the mechanisms by which this is achieved.

Our study has some limitations. As this study is purely based on quality of life, emphasis on pulmonary rehabilitation could not be given. Also, pulmonary rehabilitation, which comprises physical training program, education about disease, and nutritional, psychological, social and behavioral intervention including smoking cessation, can become a confounding factor for drug management under “WHO-GOI Guidelines-2003.” Although smoking cessation advice was given to the patients, its effect could not be evaluated due to short duration of follow-up. The study is further weakened by the relatively small number of patients and also the duration of follow-up is shorter as compared to the duration of the disease.

Despite the limitations discussed we can certainly conclude that the recommendations of WHO-GOI Guidelines for COPD-2003 are significantly favorable for management of COPD patients over conventional management.

## References

[1] Vermeire P (2002). The burden of chronic obstructive pulmonary disease. Respir Med.

[2] Jindal SK, Aggarwal AN, Gupta D (2001). A review of population studies from India to estimate national burden of chronic obstructive pulmonary disease and its association with smoking. Indian J Chest Dis Allied Sci.

[3] Sunyer J, Schwartz J, Tobias A, Macfarlane D, Garcia J, Anto JM (2000). Patients with chronic obstructive pulmonary disease are at increased risk of death associated with urban particle air pollution: A case-crossover analysis. Am J Epidemiol.

[4] Smith KR, Mehta S (2003). The burden of disease from indoor air pollution in developing countries: Comparison of estimates. Int J Hyg Environ Health.

[5] Chhabra SK, Chhabra P, Rajpal S, Gupta RK (2001). Ambient air pollution and chronic respiratory morbidity in Delhi. Arch Environ Health.

[6] Jindal SK, Gupta D, Aggarwal AN (2004). Guidelines for management of chronic obstructive pulmonary disease (COPD) in India: A guide for physicians (2003). Indian J Chest Dis Allied Sci.

[7] Quirk FH, Baveystock CM, Wilson R, Jones PW (1991). Influence of demographic and disease related factors on the degree of distress associated with symptoms and restrictions on daily living due to asthma in six countries. Eur Respir J.

[8] Jones PW, Quirk FH, Baveystock CM, Littlejohns P (1992). A self-complete measure of health status for chronic airflow limitation: The St. George's Respiratory Questionnaire. Am Rev Respir Dis.

